# Investigating Biochemical and Histopathological Responses between Raspberries and *Aculeastrum americanum*

**DOI:** 10.3390/jof9030337

**Published:** 2023-03-09

**Authors:** Márcia Gonçalves Dias, Marcel Bellato Spósito, Magda Andréia Tessmer, Beatriz Appezzato-da-Glória

**Affiliations:** 1 Biological Sciences Department, Luiz de Queiroz College of Agriculture, University of São Paulo, Piracicaba 13418-900, SP, Brazil; 2 Crop Science Department, Luiz de Queiroz College of Agriculture, University of São Paulo, Piracicaba 13418-900, SP, Brazil

**Keywords:** late leaf rust, *Pucciniastrum americanum*, *Rubus idaeus*, *Rubus occidentalis*, *Rubus niveus*, *Thekopsora americana*

## Abstract

Late leaf rust is a fungal disease in raspberries caused by *Aculeastrum americanum* (Farl.) M. Scholler U. Braun (syn. *Thekopsora americana* (Farl.) Aime McTaggart) leading to early defoliation and yield losses. Red raspberries (*Rubus idaeus* L.) are susceptible to this pathogen, although this susceptibility varies among cultivars. In contrast, black raspberries were previously reported to be more resistant (*Rubus occidentalis* L.) and immune (*Rubus niveus* Thunb.) to this pathogen, raising their importance in plant breeding programs. However, what features make them respond differently to the same pathogen? In this study, we characterize for the first time the pre- and post-formed structural and biochemical defense mechanisms of *R. idaeus* cv. Autumn Bliss, *R. occidentalis* and *R. niveus*. Ultrastructural and histopathological analyses were used to uncover the interactions between these raspberries and *A. americanum*. The ultrastructural results indicate that the pathogen germinates on both leaf surfaces but can only form appressoria on the stomata. Although the three raspberry species were infected and colonized by *A. americanum*, a clear difference in susceptibility was observed between them. A compact mesophyll, pre- and post-formed phenolic compounds, and post-formed pectic compounds were the main plant defense mechanisms against fungal colonization. These findings provide new information about raspberries’ defense mechanisms in response to *A. americanum* and elucidate the interactions occurring in these pathosystems.

## 1. Introduction

Late leaf rust, caused by *Aculeastrum americanum* (Farl.) M. Scholler & U. Braun (syn. *Thekopsora americana* (Farl.) Aime & McTaggart), affects red and purple raspberries [1,2]. The disease is hard to control and has garnered attention after outbreaks in North American orchards [1,3]. It has also been reported as a concern in Argentina, Brazil, Chile and Portugal [4,5,6,7,8].

The main symptoms are powdery yellow spots, which correspond to reproductive structures called uredinia and are found in all aerial parts of the infected plants [1,9]. Symptomatic fruits become unfit for sale, and leaves may drop prematurely, causing severe yield losses [1,10,11]. These are some outcomes of plant tissue colonization that lead to structural and physiological responses during the plant–pathogen interaction. However, it is still unknown which defense mechanisms raspberries deploy against the colonization process of *A. americanum*.

Commercial raspberries (*Rubus idaeus* L.) are classified as susceptible to late leaf rust, although this susceptibility varies among cultivars [12,13]. In contrast, black raspberries, such as *Rubus occidentalis* L. and *Rubus niveus* Thunb. are immune to this pathogen [1]. Nevertheless, late leaf rust has been documented in *R. occidentalis* [14,15,16].

Raspberries are economically relevant crops in almost all continents [17,18], thus diseases affecting these plants can not be neglected. Histopathological and biochemical studies have allowed an understanding of the important pathosystems [19,20,21,22,23,24] and have shed light on studies of the disease cycle and its epidemiology [25,26,27].

Since no histopathological investigations have yet been performed on the processes by which *A. americanum* infects and colonizes raspberries, this work seeks to elucidate whether there are differences in the biochemical traits and leaf anatomy between red and black raspberries which may hinder or delay infection by *A. americanum* in black raspberry leaves. This study focuses on uncovering the pre- and post-formed defense mechanisms in red and black raspberries.

## 2. Materials and Methods

### 2.1. Plant Material

Raspberry plants of *R. idaeus* cv. Autumn Bliss, *R. occidentalis* and *R. niveus* were grown in 7 L pots with a commercial peat substrate (Agrolink Biogrow, VDK) and kept in a greenhouse. The water was supplied by drip irrigation, and slow-release fertilizer was added every eight months at 5 g L^–1^ (N:P:K 15–09–12, Osmocote Plus^®^, Bloomington, IN, USA).

### 2.2. Biochemical Analyses

The fully expanded leaves of five different plants of each raspberry species were collected and used for the following biochemical estimations: total phenolic compounds [28], total flavonoids [29], proanthocyanidins [30,31], antioxidant activity measured by the DPPH (Diphenylpicrylhydrazyl) method [32,33], chlorophyll a, chlorophyll b, total chlorophyll, and carotenoids [34,35].

### 2.3. Light Microscopy (LM): Qualitative and Quantitative Analyses

Five healthy fully expanded leaves per species collected from different plants were sampled for qualitative and quantitative analysis of the anatomical structure. Samples were collected from the middle third of the terminal leaflet to maintain standardization (*n* = 5). The following anatomical traits were measured: total leaf thickness, cuticle thickness, the height of the epidermal cells on both leaflet surfaces, total mesophyll thickness, thickness of the palisade and spongy parenchyma, and the area of the intercellular space. Three measurements were conducted on five cross-sections per leaflet to obtain the average for each trait in each raspberry species. 

For determining the anatomical structure, 1 cm^2^ fragments of each sample were fixed for 48 h in formalin-ferrous sulfate for detection of phenolics [36] or in Karnovsky’s solution [37], subjected to a vacuum pump to remove the air from the intercellular spaces, and dehydrated in an increasing ethyl series up to 100% ethanol and infiltrated in hydroxy-ethyl-methacrylate (Leica Historesin, Heraeus Kulzer, Hanau, Germany). The obtained blocks were sectioned in a rotary microtome (Leica RM2245, Leica Biosystems, Heidelberg, Germany) at 5 μm thickness. Cross-sections from samples fixed in Karnovsky’s solution were stained with 0.05% toluidine blue (pH 4.5) [38] and mounted in water for structural analyses, or stained with Sudan IV [39] to detect lipids to delimit the cuticle. Cross-sections fixed in formalin-ferrous sulfate were mounted on slides in water for detection of the phenolic compounds.

To analyze the stomatal index (SI) and the morphometry of the stomata, five terminal leaflets were oven-dried at 37 °C for 20 min, then 1 cm^2^ of the abaxial surface in the middle third of the leaflet was shaved under a stereomicroscope using sharp bent tweezers to remove the non-glandular trichomes. These samples were submerged in a 5% (*w/v*) sodium hydroxide aqueous solution for 30 min, rinsed in distilled water and placed in a 20% (*v/v*) sodium hypochlorite solution until complete decolorization. The fragments were rinsed in distilled water, stained with Alcian Blue-Safranin O (9:1 *v/v*) [40], mounted in 50% glycerol gelatin and analyzed under the microscope. The SI was determined by counting all the stomata (S) and epidermal cells (E) in the microscopic view field and applying the formula (SI% = S/(S + E)) × 100 [41]. Morphometry of the stomata was performed by measuring the length and width of each stoma in the microscopic view field.

For quantification of the crystal idioblasts, 1 cm^2^ fragments sampled from the middle third of five leaflets were decolorized overnight in an ethanol/acetic acid solution (6:1 *v/v*) [42], rinsed in distilled water and mounted in 50% glycerol gelatin. The crystal idioblasts were visualized under polarized light, and the images were captured at 400× magnification. All crystals observed in the selected images were counted regardless of their size.

The images for qualitative and quantitative analysis were documented with a Leica DMLB microscope (Leica Microsystems) coupled to a digital camera (Leica DFC 310Fx). All measurements were performed in Image J Software version 1.52t [43].

### 2.4. Obtention and Maintenance of Aculeastrum americanum Inoculum

The monopustular isolate of *Aculeastrum americanum* was genetically characterized by Ribeiro and Spósito (GenBank: MW039448 corresponds to the ITS and MW039395 corresponds to the small subunit ribosomal RNA gene 18S) and inoculated in *R. idaeus* cv. Heritage for maintenance [44]. The isolate was collected in a commercial orchard in the municipality of São Bento do Sapucaí, São Paulo, Brazil (22°37′34.5″ S 45°34′50.4″ W) [27]. In every inoculation trial the viability of urediniospores was verified by a germination test in which three drops of the spore suspension were deposited in three Petri dishes and incubated for 24 h in a dark and humid chamber. Then, we added lactoglycerol to each drop and counted the germinated spores in 100 spores per drop.

In all experiments, 2-week-old spores were used to prepare a suspension at the concentration of 10^5^ urediniospores mL^−1^ in distilled water and Tween 20 (0.01%), and the spores’ viability was evaluated [44]. The leaves were inoculated by spraying the suspension on the entire leaf blade or by depositing drops (50 μL) on delimited areas. Leaves sprayed with distilled water were considered as the control. After inoculation, the plants were kept at 24 °C in a dark and humid chamber for 24 h, and then all plants were placed in a greenhouse until sample collection.

### 2.5. Histopathology of the Interaction between Raspberries and Aculeastrum americanum

#### 2.5.1. Scanning Electron Microscopy (SEM)

Terminal leaflets of fully expanded leaves collected from four plants of each raspberry species were used for the surface analysis. The inoculations were performed by adding spore suspension drops or distilled water to a delimited area on the leaflets’ abaxial or adaxial surface. The samples were collected 6, 12, 24, 36 h and 14 days after inoculation from three leaves per period. This experiment was performed three times. Inoculated and healthy samples were fixed in Karnovsky’s solution [37] for 48 h and subsequently dehydrated in an ethanolic series [19]. Next, they were critical-point-dried [45] (Baltec EM CPD 300), mounted on aluminum stubs and coated with a thin layer of gold (30–40 nm) by a sputter coater (Balzers, model SCD 050). The analyses and electron micrographs were performed using a scanning electron microscope (model JEOL JSM-IT300LV) operated at 15–20 kV. The images were coloured using Adobe Photoshop 2020.

#### 2.5.2. Light Microscopy (LM)

Fully expanded leaves from the three raspberry species were inoculated on the abaxial surface with an *A. americanum* spore solution or distilled water as described in Section 4. The samples were collected 14 days after inoculation (dai), and the lesions were immediately documented using a Leica DFC295 camera coupled to a Leica M205 C stereoscopy microscope. Afterwards, fragments of the samples were fixed in Karnovsky’s solution [37] for 48 h, dehydrated and infiltrated in hydroxyethyl methacrylate [19]. The samples were transversally sectioned to 5–7 μm using the rotary microtome described previously. The sections were stained with 0.05% toluidine blue (pH 4.5) [38] and mounted on slides in water. When required, other slides were stained with ruthenium red to confirm the presence of pectin and acidic mucilage [36]. Images were captured as described in Section 2.3.

### 2.6. Statistical Analyses

The statistical significance of the biochemical and anatomical traits was determined by one-way ANOVA followed by Tukey’s post-hoc test (*p* < 0.05) using RStudio software 2018 version 1.2.5033 [46].

## 3. Results

### 3.1. Characterization of Pre-Formed Defense Mechanisms in Raspberries

**Figure 1:**
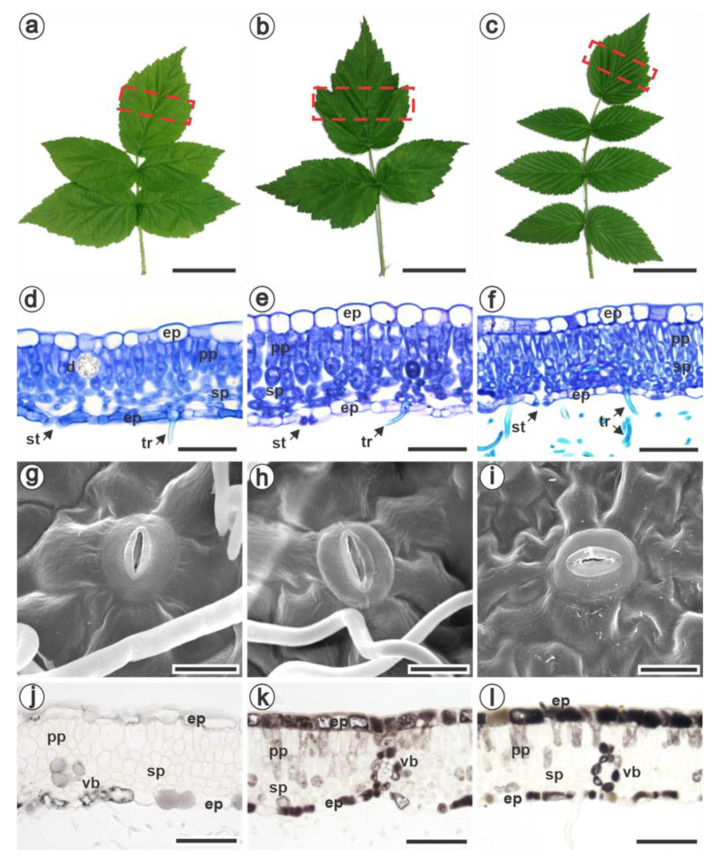
Mature leaves of *Rubus idaeus* cv. Autumn Bliss (**a**), *R. occidentalis* (**b**), and *R. niveus* (**c**). The dotted box on the terminal leaflet indicates the analyzed sector. Cross-sections of the leaflets’ lamina were stained with toluidine blue (**d**–**f**) and fixed in formalin-ferrous sulphate (**j**–**l**). (**d**–**f**) The uniseriate epidermis with larger cells on the adaxial face and the dorsiventral mesophyll with one (**d**,**e**) and two (**f**) layers of palisade parenchyma. (**g**–**i**) Scanning electron micrographs of anomocytic stomata. (**j**–**l**) Phenolic compounds (brown color) detected in epidermal, mesophyll and vascular bundle cells in all species, with lower intensity in *R. idaeus* (**j**). ep, epidermis; pp, palisade parenchyma; sp, spongy parenchyma; d, druse; st, stoma; tr, trichomes; vb, vascular bundle. Bars: (**a**–**c**): 5 cm; (**d**–**f**, **k**–**l**): 50 µm; (**g**–**i**): 10 µm; **j**: 60 µm.

The pre-formed biochemical compounds and structural defense mechanisms were investigated in red and black raspberries. The leaves showed variation in their morphology, although all three species presented compound leaves with toothed margins (Figure 1a–c). In general, *R. idaeus* cv. Autumn Bliss leaves were pentafoliolate (Figure 1a), despite being observed to be trifoliate leaves. *R. occidentalis* had trifoliate leaves (Figure 1b), while *R. niveus* had imparipinnate leaves (Figure 1c).

The cross-sections of the terminal leaflets showed a uniseriate epidermis on both surfaces, with larger cells on the adaxial face (Figure 1d–f). The cuticle thickness was higher in *R. niveus* on the adaxial surface than in the other two species (Table 1). The epidermal cells were smaller in *R. niveus* than in the other species on both leaf surfaces (Table 1, Figure 1f). The three raspberries were hypostomatic with anomocytic stomata slightly positioned above the level of the epidermis (Figure 1g–i). The width and length of the stomata were smaller in *R. niveus* than in *R. idaeus* and *R. occidentalis*, which presented similar values (Table 1).

Although numerous stomata were observed in all three species, the stomatal index was higher for *R. niveus*, which was statistically different from *R. idaeus* (Table 1). In all studied species, both epidermises showed trichomes, which were more frequent on the abaxial side. The mesophyll was dorsiventral, and its thickness did not differ among the species (Figure 1d–f, Table 1). It was more compact in *R. niveus* because of the observed two layers of palisade parenchyma, the lower spongy parenchyma thickness and almost no intercellular space (Figure 1f, Table 1). In general, *R. idaeus* and *R. occidentalis* had one layer of palisade parenchyma (Figure 1d–e), while *R. occidentalis* had a greater intercellular space than the other two species (Table 1). Idioblasts containing crystals of the druse type were frequently found in all species along the mesophyll (Figure 1d) and the midrib. Interestingly, *R. occidentalis* presented fewer druses than the other two raspberries (Table 1). Phenolic compounds were detected in the epidermal, mesophyll and vascular bundle cells in all species. However, the reaction intensity was lower in *R. idaeus* (Figure 1j) than in black raspberries (Figure 1k–l).

The observation (Figure 1j–l) that black raspberries had more constitutive phenolic compounds than *R. idaeus* was confirmed by quantitative analyses. Indeed, *R. occidentalis* and *R. niveus* presented twice as many total phenolics than *R. idaeus* (Table 1). Likewise, the proanthocyanidins stood out in these species, with higher values for *R. occidentalis* (1.20 ± 0.02) and *R. niveus* (0.87 ± 0.02). Once more, the antioxidant capacity determined by the DPPH method showed higher percentages for black raspberries (Table 1). Interestingly, the total flavonoid content was three times lower in *R. occidentalis* and *R. idaeus* compared with *R. niveus* (Table 1). The chlorophylls a, b and, consequently, the total chlorophyll, were significantly higher for *R. niveus* and were not different between *R. occidentalis* and *R. idaeus*. The same was observed for total carotenoids (Table 1). Overall, black raspberries presented the highest values for the studied biochemical traits.

### 3.2. Ultrastructural and Histopathology of the Interaction between Raspberries and Aculeastrum americanum

**Figure 2:**
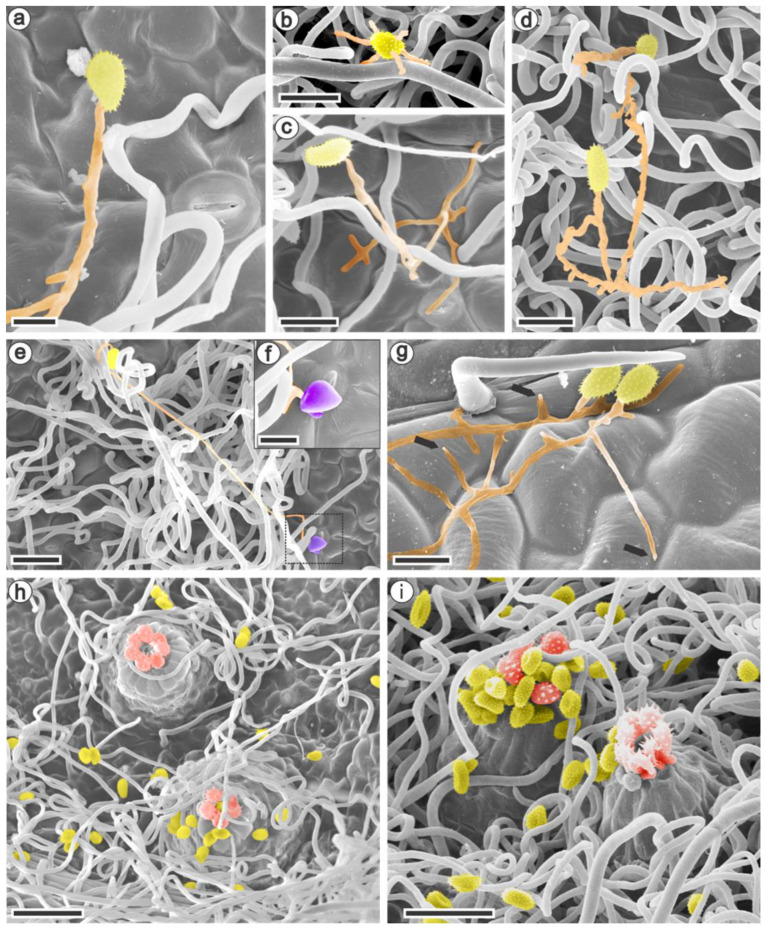
Scanning electron micrographs of the adaxial (**g**) and abaxial leaf surfaces (**a**–**f**,**h**–**i**) of *Rubus idaeus* cv. Autumn Bliss (**c**,**e**,**f**,**h**), *R. occidentalis* (**a**,**b**,**g**,**i**), and *R. niveus* (**d**) at 6 (**a**), 12 (**b**), 24 (**g**), 36 (**c**–**f**) hours, and 14 (**h**–**i**) days after inoculation with *Aculeastrum americanum*. (**a**,**b**) Urediniospores emitting one (**a**) or several (**b**) germ tubes. (**c**) Branched germ tube. (**d**) Urediniospores germinated on the trichomes in *R. niveus*. (**e**) Appressorium formed over a stoma (dotted box) showed in detail in (**f**). (**g**) Germinated urediniospores without the formation of an appressorium at the end of the germ tubes (arrows). (**h**,**i**) Satellite uredia with ostiolar cells that delimit the opening through which the urediniospores are released. *A. americanum* structures were artificially stained using Adobe Photoshop 2020^®^. Urediniospore, yellow; germ tube, orange; appressorium, purple; uredia ostiolar cells, red. Bars: (**a**,**f**): 10 µm; (**b**–**d**,**g**): 20 µm; (**e**): 30 µm; (**h**–**i**): 50 µm.

Scanning electron microscopy analysis showed the first interactions between the raspberry species and *Aculeastrum americanum*. The pathogen viability evaluated in vitro had a range of 38% to 65% germination. The urediniospores inoculated on the abaxial leaf surfaces and observed at 6 h after inoculation (hai) germinated and formed a single (Figure 2a) or multiple germ tubes (Figure 2b). Although the germ tubes could reach lengths greater than 100 µm, they had a medium to long elongation (10–100 µm) overall. It was noted that the germ tube branched in different directions when in contact with the epidermis (Figure 2c,g). Despite the urediniospore germination that occurred in *R. niveus* (Figure 2d), the formation of an appressorium was observed only at 12 and 36 hai in *R. occidentalis* and *R. idaeus* (Figure 2e,f).

To confirm that penetration occurred only via the stomata, the leaves’ adaxial surfaces were inoculated with *A. americanum* urediniospores and analyzed at 24 hai. Similar to the abaxial surface, the spores germinated, forming elongated and branched germ tubes, but no appressorium was observed (Figure 2g). The late leaf rust latency period was 7–10 days for all plants, when satellite uredinia (Figure 2h–i) emerged on the leaves’ abaxial surface with the same morphological features in all the raspberry species.

**Figure 3:**
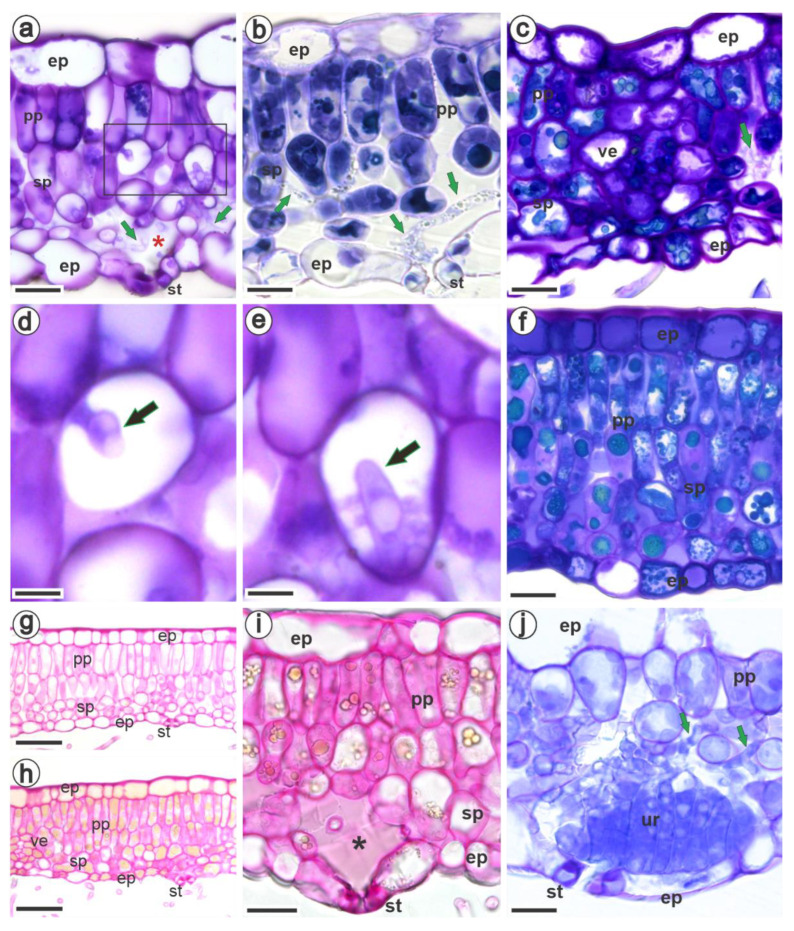
Cross-sections of *Rubus idaeus* cv. Autumn Bliss (**a**,**d**,**e**,**j**), *R. occidentalis* (**b**,**i**), and *R. niveus* (**c**,**f**,**g**,**h**) leaves 14 days after inoculation with *Aculeastrum americanum*, stained with toluidine blue (**a**–**f**,**j**) or with ruthenium red (**g**–**i**). (**a**–**c**) Hyphae in the intercellular space of the spongy parenchyma (arrows), especially in the substomatal chamber. (**d**,**e**) Detail of the black frame in ‘a’ showing the haustorium (black arrow) inside the spongy parenchyma cell. The mesophyll cells accumulated more phenols in infected areas (**f**,**h**,**i**) than in uninfected areas of the same leaf lamina (**g**); black raspberries also exhibited intercellular spaces filled with pectic substances (**f**,**i**). (**j**) Initial stages of uredinium formation in the substomatal chamber. Phenols are shown as intense blue (**a**,**b**) or greenish-blue (**c**,**f**) dots stained with toluidine blue, and yellow dots stained by ruthenium red (**h**,**i**). Pectic substances were stained purple by toluidine blue (**f**) and pink by ruthenium red (**h**,**i**). ep, epidermis; pp, palisade parenchyma; sp, spongy parenchyma; ve, vein; st, stoma; ur, uredinia; *, substomatal chamber. Bars: (**a**,**i**): 20 µm, (**b**): 12 µm, (**c**,**f**,**j**): 15 µm, (**d**–**e**): 5 µm, (**g**,**h**): 40 µm.

Mesophyll colonization in all the studied species was strictly intercellular, and the hyphae were found mainly in the spongy parenchyma (Figure 3a–c), especially in the substomatal chamber (Figure 3a,b). The pathogen grew intracellularly, forming the haustorium, consisting of the neck and haustorial body (Figure 3d,e). The mesophyll cells of all raspberries accumulated more phenols as a response to fungal colonization (Figure 3a–c,f), but particularly the black raspberries, which also showed the presence of pectic materials (Figure 3f,h,i) compared with uninfected areas (Figure 3g). The formation of uredinia began mainly in the region of the substomatal chamber (Figure 3j).

**Figure 4:**
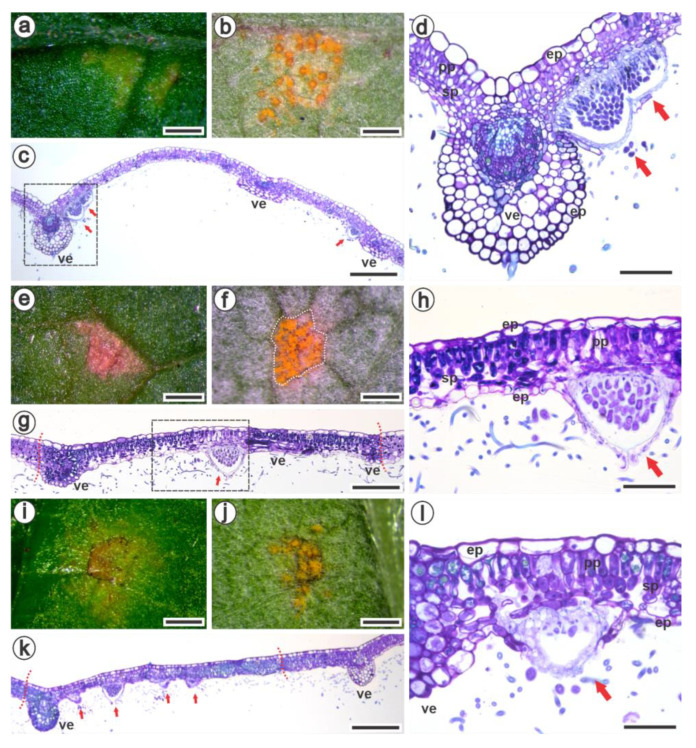
Morphoanatomy of lesions on the leaf blades of *Rubus idaeus* cv. Autumn Bliss (**a**–**d**), *R. occidentalis* (**e**–**h**) and *R. niveus* (**i**–**l**) caused by *Aculeastrum americanum* 14 days after inoculation. The visual symptoms are characterized by chlorotic (**a**) or necrotic (**e**,**i**) lesions on the adaxial surface, and correspond to the uredinia on the abaxial surface (**b**,**f**,**j**). Cross-sections of the lesioned area are shown in general view, dotted box in (**c**,**g**,**k**) and as the respective highlighted sectors (**d**,**h**,**l**). The uredinia are limited by the veins (**f**,**g**,**j**,**k**) only in *R. occidentalis* and *R. niveus*, and the mesophyll cells are intensely stained blue/greenish-blue by the accumulation of phenolic compounds (**h**,**l**). The yellow spots outside the vein correspond to released urediniospores (**j**). Pustules are indicated with arrows. ep, epidermis; pp, palisade parenchyma; sp, spongy parenchyma; ve, vein. Bars: (**a**,**b**,**e**,**f**,**i**,**j**): 0.5 mm; (**c**,**g**,**k**): 500 µm; (**d**): 100 µm; (**h**,**l**): 50 µm.

The visible symptoms observed on the adaxial surfaces (Figure 4a,e,i) 14 days after inoculation (dai) with *A. americanum* corresponded to the yellowish uredinia on the abaxial surfaces (Figure 4b,f,j). *R. idaeus* had a chlorotic lesion on the adaxial surface (Figure 4a) without apparent necrosis. In contrast, this necrotic characteristic was distinguishable in *R. occidentalis* and *R. niveus* (Figure 4e,i), since the pustules emerged on the abaxial surface. In *R. idaeus*, the necroses on the adaxial surface were identified many days afterwards. Interestingly, the pustules emerging in black raspberry leaves were limited by the leaves’ venation areoles (Figure 4f,g,j,k), in contrast to the outspread pustules found in *R. idaeus* (Figure 4b,c). During the process of image capture, the pustules sporulated, releasing the urediniospores observed outside the area surrounded by the veins (Figure 4j). The mesophyll cells of *R. occidentalis* (Figure 4g,h) and *R. niveus* (Figure 4k,l) were intensely stained blue/greenish-blue by toluidine blue because of the higher content of phenolic compounds than *R. idaeus* (Figure 4c,d).

## 4. Discussion

Structural and biochemical mechanisms have been demonstrated to help plants manage biotic and environmental stresses. This study uncovered some of these mechanisms in three raspberry species in response to the interaction with *A. americanum*, the causal agent of late leaf rust.

Previous anatomical studies on leaves from different raspberry species and varieties indicate that they are similar in most aspects, with details in some structures such as the epidermal cells and covering trichomes [47,48,49]. Because our focus was on analyzing physical barriers as potential defense mechanisms against *A. americanum*, the morphology and histochemistry of the different types of trichomes found on black raspberries could still be further investigated in the future. Even so, for all three raspberries, our results confirm the presence of very long unbranched non-glandular trichomes that are strongly tangled. According to Karley et al. [50], these trichomes negatively affect herbivore–*R. idaeus* interactions. In contrast, Wang et al. [51] suggest that the high density of trichomes contributes to the adhesion of fungal spores and produces a microclimate that favors spore germination. Indeed, even covering the raspberry leaf surface, the non-glandular trichomes are not a fully effective barrier against *A. americanum*.

The height of the cuticle coating the entire epidermis, including the stoma guard cells, was measured to check its potential role as a pre-formed resistance barrier against *A. americanum*. However, no evidence of resistance to the pathogen was found on any of the raspberry leaves. In fact, the cuticle’s role is more commonly discussed in terms of interactions in which the pathogen penetrates directly into the host [52,53].

It has been previously described that hypostomatic leaves have anomocytic stomata located above the epidermis level for *R. idaeus* varieties and *R. loganobaccus* [47,48,54]. Herein, we confirm these characteristics for *R. idaeus* cv. Autumn Bliss and, for the first time, document them for *R. occidentalis* and *R. niveus*. Among the studied raspberries, *R. niveus* has stomata with a smaller width and length, and a higher stomatal index. Having more stomata available should apparently facilitate the pathogen’s penetration [55,56,57], however, nothing that drew attention was observed for this black raspberry. Nevertheless, the morphology of the stomata is also related to the penetration process, as a small stoma might hinder the formation of an appressorium [58]. Studies addressing the composition of the wax covering the stoma and the relationship between disease severity and the stomatal index might help to uncover the role of the stomata in the *A. americanum*–raspberry pathosystem.

In all studied species, the urediniospores germinated on both leaf surfaces, yet only formed an appressorium above the stomata on the abaxial face. This strategy has been commonly observed for several rust fungi [59,60,61], although the appressorium can also be formed over epidermal cell junctions in other rust pathosystems [22,62,63]. Indeed, the medium to long germ tubes observed for *A. americanum* are a typical characteristic of urediniospores that penetrate the host through the stomata [64,65]. Moreover, the multiple germ tubes emitted by *A. americanum* may favor infection processes and increase the aggressiveness of the pathogen [66]. Another characteristic observed was the ability of the germ tube to branch. Although this event may be influenced by external conditions, such as the time of incubation and the temperature during germination [67], ramification of the germ tube was common in all experiments performed using the optimal temperature for *A. americanum*.

Following penetration, the hyphae of *A. americanum* developed in the intercellular spaces of the mesophyll, mainly in the substomatal chamber and alongside the spaces of the spongy parenchyma. The hyphae were hardly seen in the palisade parenchyma, probably because its cells are more columnar and stacked side by side, which may hamper the development and visualization of hyphae. Moreover, *R. niveus* had a compact double-layered palisade parenchyma and presented the lowest intercellular values, traits that act as physical barriers to the growth of hyphae. It has been found that thicker palisade tissue exhibits greater resistance to pathogens such as *Puccinia zoysiae* [58] and *Xanthomonas arboricola* [68].

Meanwhile, *R. occidentalis* had more intercellular spaces among the studied raspberries, presumably allowing for the development of hyphae. Interestingly, the constitutive and post-infection biochemical traits of black raspberries seem to be more effective against *A. americanum*. The intercellular spaces of infected leaves of *R. occidentalis* and *R. niveus* were filled with pectic substances, in contrast to those of *R. idaeus*. Likewise, grapevine leaves infected by *Phakopsora meliosmae-myrianthae* (syn. *P. euvitis*) deposited pectin on the cell wall and intercellular spaces [69]. Resistant genotypes of *Coffea* spp. inoculated with the rust *Hemileia vastatrix* also accumulated pectin and polysaccharides in the intercellular spaces, which was not observed in healthy and susceptible tissues [70]. It is clear from these findings that pectins are important defense mechanisms against plant pathogens, which merits further investigation.

Haustoria were frequently observed inside *R. idaeus* cells, while black raspberries accumulated more phenolic compounds after colonization by the fungus, which may have hindered their observation. In general, the mature haustorium was about 10 μm in length and had a cylindrical to allantoid shape, as described by [71].

The biochemical and histochemical results show that black raspberries have higher levels of constitutive phenolic compounds than *R. idaeus*, which might explain the more effective response to colonization by *A. americanum* in *R. occidentalis* and *R. niveus*.

Phenolics include several classes of compounds that are known for their antimicrobial activity [72,73]. Similar to phenolic compounds, black raspberries displayed higher antioxidant activity, as assessed by the DPPH method. These results corroborate the significant correlation between phenolic substances and antioxidant activity found by Oszmiański et al. [74] in *Rubus* leaves. Surprisingly, the flavonoid content was lower in *R. occidentalis*. However, it might be compensated by different phenolics such as proanthocyanidins and others not explored in this study but found in the *Rubus* species [75,76,77]. Additionally, flavonoids, proanthocyanidins and carotenoids act as antioxidants to scavenge free radicals produced during plant–pathogen interactions [78]. The staining with DAB (3,3′-diaminobenzidine) for the detection of hydrogen peroxide and with NBT (nitroblue tetrazolium) for the detection of superoxide anion did not produce any results in all raspberry species studied, even when adapting the protocols. We attributed this failure to their dense leaf pubescence promoting a nearly impenetrable layer on the abaxial leaf surface [49,75,79,80].

The epidermal and mesophyll cells of infected *R. occidentalis* and *R. niveus* accumulated polyphenols that may vastly hamper the development of haustoria. The accumulation of phenolic compounds as a response to pathogen infection has been confirmed for several pathosystems [19,20,24,69,81]. Their role is not only associated with the chemical activity but also with the structural function. Polyphenols added to the cell wall can strengthen the structure and make the formation of haustoria difficult [81]. Plants also produce lignin-like compounds by activating the phenylpropanoid pathway in response to infection [70,72,82].

It has been shown that the vascular bundle restricts pathogen colonization in Vitis vinifera cultivars [69,83]. Interestingly, it was only on the leaves of black raspberries that the uredinia appeared to have been delimited by the veins 14 days after inoculation with *A. americanum*. Nevertheless, as the infection progressed and more nutrients were needed to form uredinia, colonization also spread beyond the vascular tissues.

In conclusion, a more compact mesophyll, pre- and post-formed phenolic compounds, and post-formed pectic compounds are the main defense mechanisms found in raspberries that play a role against *A. americanum*. Although the raspberries had both pre-formed and post-formed defense mechanisms, these were not sufficient to totally contain infection and colonization by *A. americanum*. According to these results, we confirm the susceptibility of *R. idaeus* cv. Autumn Bliss to late leaf rust and show the absence of immunity to *A. americanum* for *R. occidentalis* and *R. niveus*.

## Figures and Tables

**Table 1:** Anatomical and biochemical traits of *Rubus idaeus* cv. Autumn Bliss, *R. occidentalis* and *R. niveus*. Statistically different (*p* < 0.05) values are denoted by lowercase letters within rows according to a one-way ANOVA followed by Tukey’s post-hoc test.

Anatomical Traits	*Rubus idaeus*	*Rubus occidentalis*	*Rubus niveus*
Total leaf thickness (µm)	105.30 ± 6.82 ^ab^	118.28 ± 12.91 ^a^	92.12 ± 3.84 ^b^
Adaxial cuticle thickness (µm)	0.70 ± 0.12 ^a^	0.79 ± 0.24 ^a^	1.15 ± 0.16 ^b^
Adaxial epidermal cell height (µm)	20.48 ± 3.06 ^a^	21.70 ± 1.99 ^a^	15.41 ± 1.27 ^b^
Mesophyll thickness (µm)	73.07 ± 3.27 ^a^	81.49 ± 7.84 ^a^	68.81 ± 4.15 ^a^
Palisade parenchyma thickness (µm)	37.93 ± 5.69 ^a^	36.05 ± 3.10 ^a^	45.96 ± 2.66 ^b^
Number of palisade parenchyma layers	1 (Figure 1d)	1 (Figure 1e)	1 to 2 (Figure 1f)
Spongy parenchyma thickness (µm)	35.14 ± 4.23 ^a^	45.44 ± 9.66 ^a^	22.84 ± 4.00 ^b^
Intercellular space (µm^2^)	490.78 ± 149.05 ^a^	886.05 ±156.67 ^b^	252.02 ± 41.24 ^a^
Crystal idioblasts (crystals/cm^2^)	24.57 ± 4.03 ^a^	13.48 ± 2.86 ^b^	21.93 ± 6.23 ^ab^
Abaxial epidermal cell height (µm)	18.19 ± 1.47 ^a^	14.71 ± 4.19 ^a^	9.53 ± 1.48 ^b^
Abaxial cuticle thickness (µm)	0.53 ± 0.09 ^a^	0.70 ± 0.21 ^a^	0.62 ± 0.06 ^a^
Stomata length (µm)	19.26 ± 1.16 ^a^	17.81 ± 0.83 ^a^	14.47 ± 1.06 ^b^
Stomata width (µm)	13.03 ± 1.65 ^ab^	14.58 ± 1.13 ^a^	12.30 ± 0.64 ^b^
Stomatal index	10.55 ± 1.36 ^a^	11.64 ± 2.70 ^ab^	14.12 ± 1.66 ^b^
**Biochemical traits**			
Total phenolic compounds (mg GAE g^−1^ FW)	0.40 ± 0.04 ^a^	0.92 ± 0.06 ^b^	0.76 ± 0.03 ^c^
DPPH antioxidant activity (%)	42.92 ± 0.99 ^a^	95.60 ± 1.19 ^b^	94.13 ± 0.44 ^b^
Proanthocyanidins (mg CE g^−1^ FW)	0.45 ± 0.07 ^a^	1.20 ± 0.02 ^b^	0.87 ± 0.02 ^c^
Total flavonoids (mg RE g^−1^ FW)	0.34 ± 0.02 ^a^	0.30 ± 0.01 ^a^	1.06 ± 0.08 ^b^
Chlorophyll a (mg g^−1^ FW)	2.35 ± 0.51 ^a^	2.26 ± 0.31 ^a^	3.24 ± 0.44 ^b^
Chlorophyll b (mg g^−1^ FW)	0.93 ± 0.20 ^a^	0.88 ± 0.16 ^a^	1.48 ± 0.23 ^b^
Total chlorophyll (mg g^−1^ FW)	3.28 ± 0.70 ^a^	3.16 ± 0.46 ^a^	4.72 ± 0.67 ^b^
Total carotenoids (mg g^−1^ FW)	0.77 ± 0.16 ^a^	0.84 ± 0.13 ^a^	1.12 ± 0.17 ^b^

## Data Availability

Not applicable.

## References

[1] Martin R.R., Ellis M.A., Williamson B., Williams R.N., Martin R.R., Williamson B., Williams R.N. (2017). PART I: Diseases Caused by Biotic Factors. Compendium of Raspberry and Blackberry Diseases and Pests.

[2] Scholler M., Braun U., Buchheit R., Schulte T., Bubner B. (2022). Studies on European Rust Fungi, Pucciniales: Molecular Phylogeny, Taxonomy, and Nomenclature of Miscellaneous Genera and Species in Pucciniastraceae and Coleosporiaceae. Mycol. Prog..

[3] Delisle-Houde M., Demers F., Tweddell R. (2020). Evaluation of Phytosanitary Products for the Management of Raspberry Late Leaf Rust [*Pucciniastrum americanum* (Farl.) Arthur]. Phytoprotection.

[4] Figueiredo M.B., Nogueira E.D.C., Ferrari J.T., Aparecido C.C., Hennen J.F. (2003). Ocorrência de Ferrugem Em Framboesa No Estado de São Paulo. Arq. Inst. Biol. São Paulo.

[5] Raseira M.D., Gonçalves E.D., Antunes L.E.C. (2004). Aspectos técnicos da cultura da framboeseira. https://www.embrapa.br/busca-de-publicacoes/-/publicacao/744781/aspectos-tecnicos-da-cultura-da-framboeseira.

[6] Lucero X., Wright E.R., Pérez B.A. (2008). Occurrence of Late Leaf Rust Caused by *Pucciniastrum americanum* in Red Raspberry (*Rubus idaeus*) in Buenos Aires, Córdoba, and Entre Ríos, Argentina. Plant Dis..

[7] Pio R. (2014). Cultivo de Fruteiras de Clima Temperado Em Regiões Subtropicais e Tropicais.

[8] de Oliveira P.B. (2021). Manual de Boas Práticas de Fruticultura—Framboesa.

[9] Dolan A., MacFarlane S., Jennings S.N. (2018). Pathogens in Raspberry and Other *Rubus* spp.. Raspberry: Breeding, Challenges and Advances.

[10] Hall H.K., Hummer K.E., Jamieson A.R., Jennings S.N., Weber C.A. (2009). Raspberry Breeding and Genetics. Plant Breeding Reviews.

[11] Funt R.C. (2013). Pest and Disease Management. Raspberries.

[12] Luffman M., Buszard D. (1990). A Note on the Susceptibility of Six Red Raspberry Cultivars and Tayberry to Fruit Infection by Late Yellow Rust. Phytoprotection.

[13] Nelson S. (2011). Raspberry Late Leaf Rust in Hawaii Caused by *Pucciniastrum americanum*. Plant Dis..

[14] Dodge B.O. (1923). Morphology and Host Relations of *Pucciniastrum americanum*. J. Agric. Res..

[15] Darker G.D. (1929). Cultures of *Pucciniastrum americanum* (Farlow) Arthur and *P. arcticum* (Lagerheim) Tranzschel. J. Arnold Arbor..

[16] Nickerson N.L. (1991). Late Leaf Rust. Compendium of Raspberry and Blackberry Diseases and Insects.

[17] Foster T.M., Bassil N.V., Dossett M., Leigh Worthington M., Graham J. (2019). Genetic and Genomic Resources for *Rubus* Breeding: A Roadmap for the Future. Hortic. Res..

[18] FAO FAOSTAT: Agricultural Data. http://www.fao.org/faostat/en/#data/QC.

[19] Braga Z.V., dos Santos R.F., Amorim L., Appezzato-da-Glória B. (2019). Histopathology of Infection and Colonisation of *Elsinoë ampelina* on Grapevine Leaves. Eur. J. Plant Pathol..

[20] Braga Z.V., Muniz L.F., Manarim G.R., de Aguiar C.L., Appezzato-da-Glória B. (2021). Anatomical and Biochemical Changes in Leaves of *Vitis labrusca* L. Cv. Niagara Rosada in Response to Infection by *Elsinoë ampelina* Shear. Braz. J. Bot..

[21] Primiano I.V., Loehrer M., Amorim L., Schaffrath U. (2017). Asian Grapevine Leaf Rust Caused by *Phakopsora euvitis*: An Important Disease in Brazil. Plant Pathol..

[22] Rasera J.B., Amorim L., Marques J.P.R., Soares M.K.M., Appezzato-da-Glória B. (2019). Histopathological Evidences of Early Grapevine Leaf Senescence Caused by *Phakopsora euvitis* Colonisation. Physiol. Mol. Plant Pathol..

[23] Alves R.F., Marques J.P.R., Appezzato-da-Glória B., Spósito M.B. (2021). Process of Infection and Colonization of *Pseudocercospora kaki* in Persimmon Leaves. J. Phytopathol..

[24] Marques J.P.R., Cia M.C., Batista de Andrade Granato A., Muniz L.F., Appezzato-da-Glória B., Camargo L.E.A. (2022). Histopathology of the Shoot Apex of Sugarcane Colonized by *Leifsonia xyli* subsp. xyli. Phytopathology.

[25] Nogueira Júnior A.F., Ribeiro R.V., Appezzato-da-Glória B., Soares M.K.M., Rasera J.B., Amorim L. (2017). *Phakopsora euvitis* Causes Unusual Damage to Leaves and Modifies Carbohydrate Metabolism in Grapevine. Front. Plant Sci..

[26] Boufleur T., Morales J.V.P., Martins T.V., Gonçalves M.P., Massola N.S., Amorim L. (2022). A Diagnostic Guide for Myrtle Rust. Plant Health Prog..

[27] Dias M.G., Ribeiro R.R., de A. Barbosa C.M., de Jesus J.M., Spósito M.B. (2022). Diagrammatic Scale for Improved Late Leaf Rust Severity Assessments in Raspberry Leaves. Can. J. Plant Pathol..

[28] Singleton V.L., Rossi J.A. (1965). Colorimetry of Total Phenolics with Phosphomolybdic-Phosphotungstic Acid Reagents. Am. J. Enol. Vitic..

[29] Mabry T.J., Markham K.R., Thomas M.B. (1970). Reagents and Procedures for the Ultraviolet Spectral Analysis of Flavonoids. The Systematic Identification of Flavonoids.

[30] Broadhurst R.B., Jones W.T. (1978). Analysis of Condensed Tannins Using Acidified Vanillin. J. Sci. Food Agric..

[31] Nakamura Y., Tsuji S., Tonogai Y. (2003). Analysis of Proanthocyanidins in Grape Seed Extracts, Health Foods and Grape Seed Oils. J. Health Sci..

[32] Molyneux P. (2004). The Use of the Stable Free Radical Diphenylpicrylhydrazyl (DPPH) for Estimating Antioxidant Activity. Songklanakarin J. Sci. Technol..

[33] Sharma O.P., Bhat T.K. (2009). DPPH Antioxidant Assay Revisited. Food Chem..

[34] Hiscox J.D., Israelstam G.F. (1979). A Method for the Extraction of Chlorophyll from Leaf Tissue without Maceration. Can. J. Bot..

[35] Wellburn A.R. (1994). The Spectral Determination of Chlorophylls a and b, as Well as Total Carotenoids, Using Various Solvents with Spectrophotometers of Different Resolution. J. Plant Physiol..

[36] Johansen D.A. (1940). Plant Microtechnique.

[37] Karnovsky M.J. (1965). A Formaldehyde–Glutaraldehyde Fixative of High Osmolality for Use in Electron Microscopy. J. Cell Biol..

[38] Sakai W.S. (1973). Simple Method for Differential Staining of Paraffin Embedded Plant Material Using Toluidine Blue O. Stain Technol..

[39] Jensen W.A. (1962). Botanical Histochemistry: Principles and Practice.

[40] Luque R., Sousa H.C.D., Kraus J.E. (1996). Métodos de Coloração de Roeser (1972): Modificado—e Kropp (1972) Visando a Substituição Do Azul de Astra Por Azul de Alcião 8GS Ou 8GX. Acta Bot. Bras..

[41] Paul V., Pandey R., Singh M.P. (2017). Measurements of Stomatal Density and Stomatal Index on Leaf/Plant Surfaces. Physiological Techniques to Analyze the Impact of Climate Change on Crop Plants.

[42] Hoefle C., Loehrer M., Schaffrath U., Frank M., Schultheiss H., Hückelhoven R. (2009). Transgenic Suppression of Cell Death Limits Penetration Success of the Soybean Rust Fungus *Phakopsora pachyrhizi* into Epidermal Cells of Barley. Phytopathology.

[43] Rasband W.S. (2018). Image J. https://imagej.nih.gov/ij/index.html.

[44] Ribeiro R.R., Spósito M.B. (2022). Interference of Late Rust Associated with Water Deficit in the Primary Metabolism of Raspberries. Eur. J. Plant Pathol..

[45] Horridge G.A., Tamm S.L. (1969). Critical Point Drying for Scanning Electron Microscopy Study of Cilliar Motion. Science.

[46] (2018). RStudio Team RStudio: Integrated Development for R. http://www.rstudio.com.

[47] Fell K.R., Rowson J.M. (1956). Anatomical Studies in the Genus *Rubus*: Part I. The Anatomy of the Leaf of *Rubus idaeus* L.. J. Pharm. Pharmacol..

[48] Fell K.R., Rowson J.M. (1961). Anatomical Studies in the Genus *Rubus*: Part IV. Anatomical Variations in the Leaves of Cultivated Varieties of *R. idaeus* L. and *R. loganobaccus* LH Bailey, and of Certain Species of Bramble. J. Pharm. Pharmacol..

[49] Tomaszewski D., Zieliński J., Gawlak M. (2014). Foliar Indumentum in Central-European *Rubus* Species (Rosaceae) and Its Contribution to the Systematics of the Group. Nord. J. Bot..

[50] Karley A.J., Mitchell C., Brookes C., McNicol J., O’Neill T., Roberts H., Graham J., Johnson S.N. (2016). Exploiting Physical Defence Traits for Crop Protection: Leaf Trichomes of *Rubus idaeus* Have Deterrent Effects on Spider Mites but Not Aphids: Differential Effects of Leaf Trichomes on Two Herbivores of *Rubus idaeus*. Ann. Appl. Biol..

[51] Wang Y., Zeng J., Xia X., Xu Y., Sun J., Gu J., Sun H., Lei H., Chen F., Jiang J. (2020). Comparative Analysis of Leaf Trichomes, Epidermal Wax And Defense Enzymes Activities in Response to *Puccinia horiana* in *Chrysanthemum* and *Ajania* Species. Hortic. Plant J..

[52] Mendgen K., Deising H. (1993). Infection Structures of Fungal Plant Pathogens—A Cytological and Physiological Evaluation. New Phytol..

[53] Bettgenhaeuser J., Gilbert B., Ayliffe M., Moscou M.J. (2014). Nonhost Resistance to Rust Pathogens—A Continuation of Continua. Front. Plant Sci..

[54] Fell K.R., Rowson J.M. (1960). Anatomical Studies in the Genus *Rubus*: Part III. The Anatomy of the Leaf of *Rubus loganobaccus* LH Bailey. J. Pharm. Pharmacol..

[55] Avelino J., Willocquet L., Savary S. (2004). Effects of Crop Management Patterns on Coffee Rust Epidemics. Plant Pathol..

[56] Perera M.F., Bertani R.P., Arias M.E., Hechavarría M.D.L.L.L.O., Navarro M.D.L.Á.Z., Debes M.A., Luque A.C., Cuenya M.I., Rojas R.A., Castagnaro A.P. (2020). Morphological and Molecular Characterization of *Puccinia kuehnii*, the Causal Agent of Sugarcane Orange Rust in Cuba. Sci. Agric..

[57] Muir C.D. (2020). A Stomatal Model of Anatomical Tradeoffs Between Gas Exchange and Pathogen Colonization. Front. Plant Sci..

[58] Li M., Li W., Sun Y., Mao P., Qi X., Wang Y. (2018). Analysis of Leaf Tissue Structures between Rust-Resistant and Rust-Susceptible Zoysia Grass (*Zoysia japonica*). Acta Physiol. Plant..

[59] Kolmer J.A., Ordonez M.E., Groth J.V. The Rust Fungi. In eLS; John Wiley & Sons, Ltd., Chichester, UK, 2009; ISBN 978-0-470-01617-6.

[60] Solanki S., Ameen G., Borowicz P., Brueggeman R.S. (2019). Shedding Light on Penetration of Cereal Host Stomata by Wheat Stem Rust Using Improved Methodology. Sci. Rep..

[61] Duplessis S., Lorrain C., Petre B., Figueroa M., Dodds P.N., Aime M.C. (2021). Host Adaptation and Virulence in Heteroecious Rust Fungi. Annu. Rev. Phytopathol..

[62] Goellner K., Loehrer M., Langenbach C., Conrath U., Koch E., Schaffrath U. (2010). *Phakopsora pachyrhizi*, the Causal Agent of Asian Soybean Rust. Mol. Plant Pathol..

[63] Yong W.T.L., Ades P.K., Tibbits J.F.G., Bossinger G., Runa F.A., Sandhu K.S., Taylor P.W.J. (2019). Disease Cycle of *Austropuccinia psidii* on *Eucalyptus globulus* and *Eucalyptus obliqua* Leaves of Different Rust Response Phenotypes. Plant Pathol..

[64] Hunt P. (1968). Cuticular Penetration by Germinating Uredospores. Trans. Br. Mycol. Soc..

[65] Adendorff R., Rijkenberg F.H.J. (2000). Scanning Electron Microscopy of Direct Host Leaf Penetration by Urediospore-Derived Infection Structures of *Phakopsora apoda*. Mycol. Res..

[66] Babu A.M., Philip T., Kariappa B.K., Kamble C.K. (2009). Scanning Electron Microscopy of the Infection Process of *Cercospora henningsii* on Cassava Leaves. J. Phytopathol..

[67] Minchio C.A., Fantin L.H., de Oliveira K.B., Rocha J.A., Canteri M.G. (2017). Morphological Changes of the Uediniospore of *Puccinia Kuehnii* Germ Tube in Function of Temperature. Agron. Sci. Biotechnol..

[68] Yang H., Han S., He D., Jiang S., Cao G., Wan X., Chen L., Xiao J., Zhu P. (2021). Resistance Evaluation of Walnut (*Juglans* spp.) against *Xanthomonas arboricola* and the Correlation between Leaf Structure and Resistance. For. Pathol..

[69] Navarro B.L., Marques J.P.R., Appezzato-da-Glória B., Spósito M.B. (2019). Histopathology of *Phakopsora euvitis* on *Vitis vinifera*. Eur. J. Plant Pathol..

[70] Silva M.D.C., Várzea V., Guerra-Guimarães L., Azinheira H.G., Fernandez D., Petitot A.-S., Bertrand B., Lashermes P., Nicole M. (2006). Coffee Resistance to the Main Diseases: Leaf Rust and Coffee Berry Disease. Braz. J. Plant Physiol..

[71] Moss E.H. (1926). The Uredo Stage of the Pucciniastreae. Ann. Bot..

[72] Lygin A.V., Li S., Vittal R., Widholm J.M., Hartman G.L., Lozovaya V.V. (2009). The Importance of Phenolic Metabolism to Limit the Growth of *Phakopsora pachyrhizi*. Phytopathology.

[73] Cheng Y., Zhang H., Yao J., Wang X., Xu J., Han Q., Wei G., Huang L., Kang Z. (2012). Characterization of Non-Host Resistance in Broad Bean to the Wheat Stripe Rust Pathogen. BMC Plant Biol..

[74] Oszmiański J., Wojdyło A., Nowicka P., Teleszko M., Cebulak T., Wolanin M. (2015). Determination of Phenolic Compounds and Antioxidant Activity in Leaves from Wild *Rubus* L. Species. Molecules.

[75] Costea T., Vlase L., Gostin I.N., Olah N.K., Predan G.M.I. (2016). Botanical Characterization, Phytochemical Analysis and Antioxidant Activity of Indigenous Red Raspberry (*Rubus idaeus* L.) Leaves. Stud. Univ. Vasile Goldis Ser. Stiintele Vietii Life Sci. Ser..

[76] George B.P., Thangaraj P., Chandran R., Saravanan S. (2014). A Comparative Study on *in vitro* and *in vivo* Antioxidant Properties of *Rubus ellipticus* and *Rubus niveus*. Pharmacologia.

[77] Shibu Prasanth S.C.R., Chandran P. (2017). Phytochemical and Antimicrobial Analysis of Leaf Samples of Different *Rubus* Species. Int. J. ChemTech Res..

[78] Lu Y., Chen Q., Bu Y., Luo R., Hao S., Zhang J., Tian J., Yao Y. (2017). Flavonoid Accumulation Plays an Important Role in the Rust Resistance of *Malus* Plant Leaves. Front. Plant Sci..

[79] Upadhyaya M.K., Furness N.H. (1998). Primocane Morphology and Leaf Surface Characteristics of Greenhouse-Grown Red Raspberry Cultivars. HortScience.

[80] Chwil M., Kostryco M. (2020). Histochemical Assays of Secretory Trichomes and the Structure and Content of Mineral Nutrients in *Rubus idaeus* L. Leaves. Protoplasma.

[81] Rincón Barón E.J., Gutiérrez Rodríguez A.M., Guerra Sierra B.E., Matías S.E. (2020). Alteraciones Histopatológicas Causadas Por La Roya *Puccinia nakanishikii* (*Pucciniales*: Pucciniaceae) En Plantas de *Cymbopogon citratus* (Poaceae). Rev. Biol. Trop..

[82] Marques J.P.R., Hoy J.W., Appezzato-da-Glória B., Viveros A.F.G., Vieria M.L.C., Baisakh N. (2018). Sugarcane Cell Wall-Associated Defense Responses to Infection by *Sponsorium scitameneum*. Front. Plant Sci..

[83] Unger S., Büche C., Boso S., Kassemeyer H.-H. (2007). The Course of Colonization of Two Different *Vitis* Genotypes by *Plasmopara viticola* Indicates Compatible and Incompatible Host-Pathogen Interactions. Phytopathology.

